# A RXR Ligand 6-OH-11-O-Hydroxyphenanthrene with Antitumour Properties Enhances (−)-Epigallocatechin-3-gallate Activity in Three Human Breast Carcinoma Cell Lines

**DOI:** 10.1155/2014/853086

**Published:** 2014-06-11

**Authors:** Fulvia Farabegoli, Marzia Govoni, Carmen Ciavarella, Marina Orlandi, Alessio Papi

**Affiliations:** ^1^Department of Pharmacology and Biotechnology (FaBiT), University of Bologna, Via San Giacomo 14, 40126 Bologna, Italy; ^2^Department of Specialistic, Diagnostic and Experimental Medicine (DIMES), University of Bologna, Via San Giacomo 14, 40126 Bologna, Italy; ^3^Department of Specialistic, Diagnostic and Experimental Medicine (DIMES), University of Bologna, Via Massarenti 9, 40138 Bologna, Italy; ^4^Department of Biological, Geological, and Environmental Sciences, (BiGea), University of Bologna, Via Belmeloro 6, 40126 Bologna, Italy

## Abstract

(−)-Epigallocatechin-3-gallate (EGCG) and chemotherapeutic agents cotreatment can improve cytotoxicity against cancer cells. We showed that EGCG and the rexinoid 6-OH-11-O-hydroxyphenanthrene (IIF), given together, were cytotoxic toward MCF-7, MCF-7TAM, and MDA-MB-231, three breast carcinoma cell lines showing different molecular characteristics. Cell growth arrest and apoptosis were greater after EGCG and IIF cotreatment than after individual administration. Cytotoxicity was related to upregulation of 67-kDa laminin receptor (LR67), one of the principal molecular targets of EGCG, and activation of the nuclear retinoic X receptors (RXRs) pathway. Furthermore, the transcription factor Forkhead box O3 (Foxo3a), a protein able to trigger apoptosis through upregulation of genes necessary for cell death, was activated. EGCG and IIF cotreatment produced a significant nuclear import of Foxo3a from the cytoplasm in MCF-7, MCF-7TAM, and MDA-MB-231 cells. In MCF-7TAM cells only, Foxo3a nuclear localization was associated with p473AKT downregulation. For the first time we showed that when EGCG and IIF, two harmless molecules, were given together, they might increase cytotoxicity in three breast carcinoma cell lines, two of them being representative of poorly responsive breast carcinoma types.

## 1. Introduction 

Breast cancer is the most common cancer diagnosed in women worldwide: incidence rates are highest in Western Europe and lowest in Eastern and Middle Africa. Different treatment options and protocols are considered for each stage and type of breast cancer, including the use of systemic chemotherapy drugs, cytotoxic for both normal and malignant cycling cells. Resistance to selective oestrogen receptor modulators (SERMs), chiefly tamoxifen, and to chemotherapy drugs often occurs, failing the goal of long-lasting remission and exposing patients to short and long-time side effects.

(−)-Epigallocatechin-3-gallate (EGCG) is the most important catechin present in green tea, a very popular beverage consumed all over the world. Numerous* in vitro* and animal studies suggest that green tea catechins may play a role in lowering risk and upset of several chronic diseases, especially diabetes, cardiovascular diseases [1, 2], and cancer [3, 4]. Green tea catechins inhibit cancer development of modulating key cellular proteins involved in numerous signal transduction pathways and thus altering the function of genes involved in cell proliferation, invasion, angiogenesis, and apoptosis [5, 6]. The molecular targets and mechanisms by which EGCG exerts chemopreventive activities are only in part elucidated [7]. An important issue is the difference in green tea catechin concentration observed* in vitro* and* in vivo*: in animal models, the measured EGCG in the blood and tissues was in the submicromolar levels, a concentration that is effective as chemopreventive in animals treated for a long time [8]. A strategy to increase EGCG efficacy at a low concentration may be to combine EGCG with drugs, taking advantage of additive/synergistic mechanisms. Lee and coworkers demonstrated that EGCG efficacy at low concentration can be increased by the contemporary administration of all-trans-retinoic acid (ATRA) [9]. ATRA is a retinoid used in the treatment of adult and paediatric acute leukaemia (AL) since late 1980s [10]. ATRA upregulates the synthesis of laminin receptor 67 kD (LR67), which plays a key role in cell adhesion and metastatic process [11] and it is one of the molecular targets of EGCG. The binding of EGCG to LR67 was first demonstrated by plasmon surface resonance on MCF-7 and A549 cancer cells [12] and thereafter in various cancer cell lines [13, 14]. Coherently, LR67 siRNA downregulation attenuated EGCG cytotoxicity [14]. These studies demonstrated that LR67 was a pivotal molecule leading to cell death after EGCG treatment and they first pinpointed that the combination of EGCG and a synthetic retinoid could be a promising strategy to be investigated in cancer prevention and treatment.

Molecules able to activate the retinoid acid receptor (RARs) and retinoic X receptors (RXRs) pathways have been demonstrated to inhibit mammary carcinogenesis in carcinogen-treated rats and in transgenic mice [15, 16]. ATRA has been investigated in breast cancer treatment with questionable results and toxicity [17, 18]. In contrast, rexinoids showed chemopreventive effect and low toxicity [19]. RXR-selective retinoid, LGD1069 [20], or bexarotene (Targretin), suppressed both oestrogen receptor (ER) positive and ER negative breast carcinoma development with minimal toxicity [21].

6-OH-11-O-hydroxyphenanthrene (IIF) is a RXR ligand that was found to be cytotoxic to several cancer cell lines [22, 23] but nontoxic for normal cells [24]. ATRA has been successfully used in adult and paediatric leukaemia, but 14–16% of patients develop the retinoic acid syndrome, showing respiratory distress and fever [25]. For this reason, alternative molecules able to bind either RARs or RXRs are under investigation, and IIF is considered a promising candidate. We undertook a study about the administration of EGCG in combination with IIF on three breast carcinoma cell lines, differing in biomolecular characteristics: MCF-7 is an ER positive cell line, having active p53 and low level of ErbB2 and EGFR1. This cell line is typical of hormone related breast carcinoma (corresponding to 70% of breast cancers diagnosed in women), which strongly depends on oestradiol for growth. MCF-7TAM cell line was developed in our laboratory by growing MCF-7 cells in 10^−7 ^M 4-OH-hydroxytamoxifen for six months [26] and it represents a hormonal therapy resistant phenotype. MDA-MB-231 cells represent the “triple negative” phenotype of breast carcinoma (TNBC), as they lack ER*α* and ErbB2 expression and present a mutated p53 protein. We asked whether EGCG and IIF were cytotoxic when administrated together, whether LR67 high expression and RXR activation were involved in cytotoxicity, and which molecular mechanisms contributed to cell death.

## 2. Material and Methods

### 2.1. Cell Lines

MCF-7 and MDA-MB-231 were purchased from the American Type Culture Collection (Rockville, MD, USA) and maintained in E-MEM (MCF-7) or D-MEM (MDA-MB-231) supplemented with 10% foetal bovine serum (FBS), 2 mM L-glutamine, 500 U/mL penicillin, and 50 *μ*g/mL streptomycin and grown at 37°C in a humidified atmosphere with 5% CO_2_. MCF-7 cells resistant to tamoxifen (MCF-7TAM) were selected by growing MCF-7 cells in MEM medium without phenol red, containing 2 mM glutamine, 500 U/mL penicillin, 50 *μ*g/mL streptomycin, 10% FBS serum charcoal treated, and 10^−7 ^M 4-OH-Hydroxytamoxifen. A stable cell line was obtained and named as MCF-7TAM as previously described [26].

### 2.2. Reagents

EGCG, 4-OH-Hydroxy-tamoxifen, L-glutamine, Penicillin-Streptomycin, Sulforhodamine B, MEM medium without L-glutamine and phenol red, phytohaemagglutinin (PHA), 4′,6-diamidino-2-phenylindole (DAPI), 1,4-Diazabicyclo[2.2.2]octane (DABCO) and G418 were all purchased by Sigma-Aldrich, MO, USA. E-MEM, D-MEM, RPMI 1640, and FBS were purchased by Lonza Group Ltd., Basel, Switzerland. Formalin 40% was from Carlo Erba, Milano, Italy. The following antibodies were used: anti-Bax from Applied Biosystem, USA; anti-Bcl2, anti-LR67, anti-actin, anti-*γ*-tubulin and anti-mouse-FITC conjugated from Sigma-Aldrich, MO, USA; anti-AKT, anti-p473AKT, anti-Foxo3a, anti-rabbit and anti-mouse-IgG-HRP-conjugated from Thermo Scientific, IL, USA; anti rabbit-IgG-Rhodamine-conjugated from Novus Biologicals, CO, USA. Lipofectamine 2000 was purchased by Invitrogen, CA, USA.

### 2.3. Sulforhodamine B (SRB) Assay

Cell viability after drug treatments can be estimated by SRB assay: the dye binds the cell proteins and the result of the SRB assay is linear with cell number and cellular proteins. MCF-7, MDA-MB-231, and MCF-7TAM cells (10^4^/well) were grown in 96-well plates and treated with EGCG and/or IIF for 24, 48, and 72 h at different concentrations. After treatment, the cells were washed twice with PBS and fixed in 50% aqueous trichloroacetic acid (TCA) for 1 h at 4°C (25 *μ*L/well), rinsed several times with water and incubated with 50 *μ*L/well SRB solution (0.4% in 1% acetic acid) for 30 min. SRB solution in excess was washed off by 1% acetic acid. The cells were incubated in 10 mM Tris for 20 min and the absorbance of each well was measured in a microplate reader (Bio-Rad, Hercules, CA, USA) at 570 nm. The results were expressed as a percentage of treated on controls (untreated cells).

### 2.4. Synergy Evaluation

The combination index (CI) was calculated to determine the synergistic, additive, or antagonistic effects of EGCG and IIF administration to breast carcinoma cells applying a test modified by Romanelli et al. [27]. Briefly, the expected cell survival (Sexp, the product of the survival observed with drug A alone and the survival observed with drug B alone) and the observed cell survival (Sobs) for the combination of A and B were used to construct a CI according to the formula: CI = Sexp/Sobs. CI values >1, =1, or <1 indicated a synergistic, additive, or antagonistic interaction, respectively.

### 2.5. Lymphocytes Isolation

Human peripheral blood lymphocytes (PBL) were isolated from a male healthy donor and grown in RPMI 1640 medium supplemented with 10% FBS (Lonza), 2 mM glutamine, 500 U/mL penicillin, and 50 *μ*g/mL streptomycin at 37°C in a humidified atmosphere with 5% CO_2_. Lymphocytes were incubated with 40 *μ*g/mL PHA for 72 h.

### 2.6. Western Blot Analysis

The cells were treated with EGCG and/or IIF for 24 h. After treatment cells were scraped and centrifuged at 300 ×g for 10 min. The pellets were suspended in lysis buffer (20 mM Tris-HCl, pH 7.5, 0.5 mM EDTA, 0.5% Triton X-100, 5 *μ*M Na_3_VO_4_) and sonicated on ice, in the presence of protease inhibitors. Protein concentration was determined by the method of Lowry. Cell lysates (50 *μ*g of protein per lane) were size fractioned in 10% SDS-polyacrylamide before transferring to Hybond TM-C Extra (GE Healthcare, Milan, Italy) by standard protocols. Membranes were blocked for 2 h with 5% milk in transfer buffer saline (TBS) with 0.1% Tween 20 at room temperature. The membranes were incubated overnight at 4°C with the antibodies. The following antibodies were used: anti-Bax (Applied Biosystem), anti-Bcl2 (Sigma-Aldrich), anti LR67 (Sigma-Aldrich) anti AKT, and anti p473AKT (both from Thermo Scientific) dissolved in TBS-5% milk. The membranes were washed two times with TBS with 0.1% Tween 20 and they were incubated for 1 h with the respective HRP-conjugated secondary antibodies. The primary antibodies were diluted 1 : 500 and the anti-rabbit or anti-mouse HRP-conjugated secondary antibody was diluted 1 : 1000. The proteins were detected by luminol (GE Healthcare, UK). Bands were quantified by using a densitometric images analysis software (Image Master VDS, Pharmacia Biotech, Sweden). Protein loading was controlled by anti-actin (1 : 1000) or anti-*γ*-tubulin (1 : 1000) (both from Sigma-Aldrich) detection. Experiments were performed in triplicate, normalized against actin or *γ*-tubulin control, and statistically evaluated.

### 2.7. DAPI Staining

DAPI staining was performed on MCF-7, MCF-7TAM, and MDA-MB-231 cells and on PBL stimulated to proliferation by PHA in order to detect and count apoptotic nuclei. After treatments, the cells were fixed with methanol, air dried and stained with a solution of 1 *μ*g/mL DAPI in DABCO (Sigma-Aldrich), and analysed by a Nikon fluorescence microscopy equipped with a filter for DAPI. Cells with condensed or fragmented nuclei were counted on 3–5 fields of each coverslip for a total of 150–200 cells. The percentage of cells with chromatin condensation and nuclear fragmentation was determined in two samples for each treatment and compared with controls.

### 2.8. Immunofluorescence

Cells were grown on coverslips. After treatments, they were fixed with 1% formalin in PBS for 20 min at room temperature and washed twice in PBS. Cells immunostained with Foxo3a were also treated with cold absolute ethanol to improve the antibody penetration. The samples were incubated for 30 min in a blocking solution including 10% Bovine Serum Albumin (BSA) (Sigma-Aldrich) in PBS for 1h at 37°C. Primary antibodies (anti-LR67, 1 : 400 in 1% BSA in PBS or anti-Foxo3a 1:400 in 1% BSA in PBS) were incubated for 1 h at 37°C or overnight at 4°C, respectively. After washing, the samples were incubated with 1 : 800 anti-mouse-FITC conjugated secondary antibody (Sigma-Aldrich) in 1% BSA in PBS or 1 : 800 anti-rabbit-Rhodamine conjugated secondary antibody (Novus Biologicals) in 1% BSA in PBS for 1 h at 37°C, washed, air dried, and mounted in a solution 1 : 500 DAPI in DABCO and analysed by a Nikon fluorescent microscopy equipped with filters for FITC, TRITC, and DAPI.

### 2.9. Cell Transfection

We used the plasmid pCDNA3.1 expressing LR67 cDNA (pLR67, generous gift of Elda Tagliabue, Fondazione IRCCS Istituto Nazionale dei Tumori, Milano, Italy) and pCDNA3.1 plasmid vehicle, plV) for transfection experiments. MDA-MB-231 cells (5 × 10^5^ cells in 3.5 cm Petri dish) were seeded in D-MEM medium without antibiotics overnight. The cells were washed with PBS and then they were incubated in the mixture of transfection: 4 *μ*g of each plasmid (vehicle or expressing LR67 plasmid) and Lipofectamine 2000 (Invitrogen) 10 *μ*L were separately dissolved in 1 mL of D-MEM medium without antibiotics and then combined into each plate and incubated for 24 h according to the manufacturer's instructions. After 24 h, the medium was replaced with complete D-MEM and finally 800 *μ*g/mL G418 (Sigma-Aldrich) was added for positive selection of transfected cells.

### 2.10. RNA Extraction and Reverse Transcription

Total RNA was extracted from harvested cells by guanidinium-phenol-chloroform method as described by Chomczynski and Sacchi [28] and was quantified spectrophotometrically. Two *μ*g of RNA for each sample was reverse transcribed using RevertAid First Strand cDNA Synthesis Kit (Fermentas-Thermo Fisher Scientific Inc. Waltham, MA, USA) following the manufacturer's protocol.

### 2.11. Reverse Transcription-PCR (RT-PCR)

The expression of Hoxa1, Foxo3a, and GCNF gene was evaluated by RT-PCR using *β*-actin or GAPDH as internal controls of the reaction. The sequence of Hoxa1, GCNF, and GAPDH primers was designed using Primer3 online primer design tool.

Hoxa1: F5′-GGGAAAGTTGGAGAGTACGGC-3′ and R5′-CCTCAGTGGGAGGTAGTCAG-3′; *β*-actin: F5′-GGCATCGTGATGGACTCCG-3′ and R5′-GCTGGAAGGTGGACAGCGA-3′; GCNF: F5′-CTGCTCAAATGCCTCCAGAT-3′ and R5′-GGCCTCTTCCTCAAACTCCT-3′; GAPDH: F5′-GCAGGGATGATGTTCTGGAG-3′ and R5′-TGGTATCGTGGAAGGACTCATGAC-3′. Primers for Foxo3a were described in Kuo and Lin [29]: Foxo3a: F5′-CTTCAAGGATAAGGGCGACAG-3′ and R5′-TCGTCCTGGACTTCATCCAAC-3 and they were coupled to primers for *β*-actin F5′-GGCGGCACCACCATGTACCCT-3 and R5′-AGGGGCCGGACTCGTCATACT-3′ as described by Liu [30]. Annealing temperature was 58°C for Hoxa1, 55°C for Foxo3a, and 53°C for GCNF. PCR products were loaded onto a 2% agarose gel, run into an electrophoresis chamber, stained by ethidium bromide, and visualized with a UV-transilluminator. Bands were analysed by Kodak Electrophoresis Detection and Analysis System (EDAS 290) (Eastman Kodak Company, Rochester, NY, USA).

### 2.12. Quantitative Real Time PCR (qRT-PCR)

Real Time PCR analysis of cDNA was performed using a fluorescent nucleic acid dye similar to SYBR Green (SsoFast EvaGreen Supermix, BioRad Laboratories Inc., Hercule, CA, USA) in a CFX96 system (BioRad Laboratories Inc. Hercule, CA, USA).

The following couples of primers were used: LR67: F5′-GCAGCAGGAACCCACTTAGG-3′ and R5′-GGCAGCAGCAAACTTCAGC-3′; *β*-actin: F5′-GGCGGCACCACCATGTACCCT-3 and R5′-AGGGGCCGGACTCGTCATACT-3′ as described by Livak and Schmittgen [31]. Primers to quantify RXR*α*, RXR*β*, and RXR*γ* with respect to GAPDH were designed using Primer3 online primer design tool.

RXR*α* F5′-CAAGGACTGCCTGATGACA-3′ and R5′-CGACTCCACCTCATTCTCGT-3′; RXR*βγ*: F5′-GGTTTGCCAAGCTGCTGCT-3′ and R5′-CATCTCCATGAGGAAGGTGT-3′, these last two primers amplify both RXR*β* and *γ* mRNA form; primers for GAPDH were above reported.

We used the 2^−ΔΔCT^ method for relative quantification of gene expression [31]. All samples were run in triplicate in 10 *μ*L reaction volume containing 200 ng of cDNA. The thermal cycler was programmed as follows: 30 s at 95°C and 40 cycles of 5 s at 95°C and 20 s at 58°C or at 56°C for LR67 and RXRs amplification, respectively.

### 2.13. Statistical Analysis

All experiments were performed in triplicate. Statistical significance was assessed by ANOVA multiple comparison test with standard deviation (SD), as appropriate, using PRISM 5.1 (Graph Pad Software, La Jolla, CA, USA). The level for accepted statistical significance was *P* < 0.05.

## 3. Results

### 3.1. The Combination of EGCG and IIF Increased Breast Carcinoma Cell Cytotoxicity

The ability of EGCG and IIF to suppress cell proliferation was tested by SRB assay. Following incubation with 0, 25, 50, and 100 *μ*g/mL EGCG for 24, 48, and 72 h, dose-dependent cell growth suppression was observed: 50 *μ*g/mL EGCG elicited nearly 60% suppression in proliferation after 72 h treatment in MCF-7 cells. Cell growth inhibition was around 50% after 48 h in MCF-7TAM cells (Supplementary Figures 1A  and  B in Supplementary Material available online at http://dx.doi.org/10.1155/2014/853086). In contrast, EGCG treatments were less effective in MDA-MB-231 cells (Supplementary Figure 1C). IIF was administered individually at various concentrations (0, 10, 20, 30, and 40 *μ*M) and cytotoxicity >25% was detected in all the investigated cell lines at concentrations greater than 20 *μ*M (MCF-7 cells) and 30 *μ*M (MCF-7TAM and MDA-MB-231) (Supplementary Figure 1D–F) after 72 h treatment.

**Figure 1:**
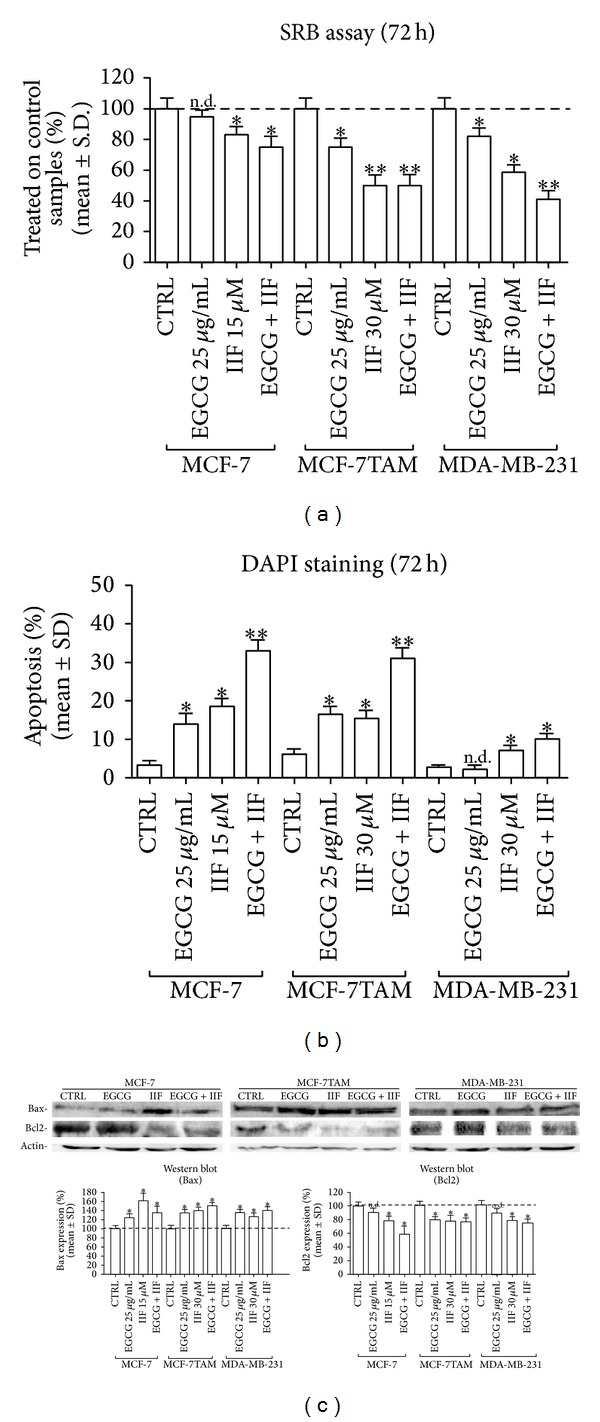
Cytotoxicity assay and apoptosis after EGCG, IIF, and EGCG + IIF treatments. (a) SRB assay after 72 h treatment. Cytotoxicity was found to increase after both individual and combined (EGCG + IIF) treatments. CTRL: untreated cells. Data shown represent the % treated on control samples of three replicas confirmed in two independent experiments. (b) DAPI staining. Apoptotic nuclei were detected by DAPI staining after 72 h incubation with EGCG, IIF, and EGCG + IIF at the indicated concentrations. The number of apoptotic nuclei for field was recorded by epifluorescence microscope and the average percentage was calculated. (c) Apoptosis Bax and Bcl2 detection in control (CTRL) and treated samples after 24 h incubation with EGCG, IIF, and EGCG + IIF at the indicated concentrations. Significant increase in Bax expression and decrease in Bcl2 expression were detected. *P < 0.05; **P < 0.01. n.d.: not detected as significant.

We used suboptimal EGCG and IIF concentrations able to give a modest cytotoxicity individually (Figure 1(a)) to investigate the presence of additive or synergistic effects. The combination 25 *μ*g/mL EGCG + 15 *μ*M IIF (MCF-7) or 30 *μ*M IIF (MCF-7TAM and MDA-MB-231) resulted in additive/synergistic effects in MCF-7, MCF-7TAM, and MDA-MB-231 cells after 48 h treatments. Antagonist effect was found in MCF-7TAM cells after 72 h treatment (Supplementary Table 1).

### 3.2. Apoptosis in Breast Carcinoma Cells and in Human Lymphocytes from Peripheral Blood Stimulated with PHA after EGCG and IIF Treatments

Cell growth arrest was also associated with cell death by apoptosis. MCF-7, MCF-7TAM, and MDA-MB-231 cells were stained with DAPI, a fluorescent molecule that binds strongly to A-T rich regions of DNA and it enables apoptotic nuclei to be visualized and counted. Cell death increased after EGCG and IIF treatments for 72 h with a sequence of events typical for apoptosis: cell shrinkage, nuclear fragmentation, altered cytoskeleton architecture, and sudden cell disruption. The percentage of apoptotic nuclei was significantly higher in EGCG + IIF treated MCF-7 and MCF-7TAM cells with respect to controls. In contrast, a few apoptotic nuclei were detected in MDA-MB-231 cells at any time of treatment (Figure 1(b)).

A more accurate analysis of the intrinsic pathway of apoptosis was carried out by evaluating Bcl2 and Bax proteins by Western blot. We found that incubation of MCF-7, MCF-7TAM, and MDA-MB-231 cells with EGCG + IIF for 24 h resulted in Bcl2 expression decrease and Bax increase (Figure 1(c)). These data suggested that EGCG and IIF induced apoptosis, possibly via mitochondrial death pathway after either individual or combined treatment. EGCG only failed to decrease Bcl2 expression in MCF-7 and MDA-MB-231 cells (Figure 1(c)).

Lymphocytes are very sensitive to toxic molecules and chemicals and they have often served as a model of clinical toxicity of drugs. For this reason, we isolated human normal lymphocytes from peripheral blood and, after stimulation with PHA for 72 h, we treated them with EGCG and IIF for 72 h. As shown in Supplementary Figure 2, apoptosis was not significantly detected.

### 3.3. Effect of EGCG and IIF Treatments on LR67 Expression

We investigated whether EGCG and IIF, administered individually and in combination, could vary the expression of their molecular targets LR67 (EGCG) and RXRs (IIF), respectively, and whether the resulting cytotoxicity could be assigned to increase in molecular targets availability.

**Figure 2:**
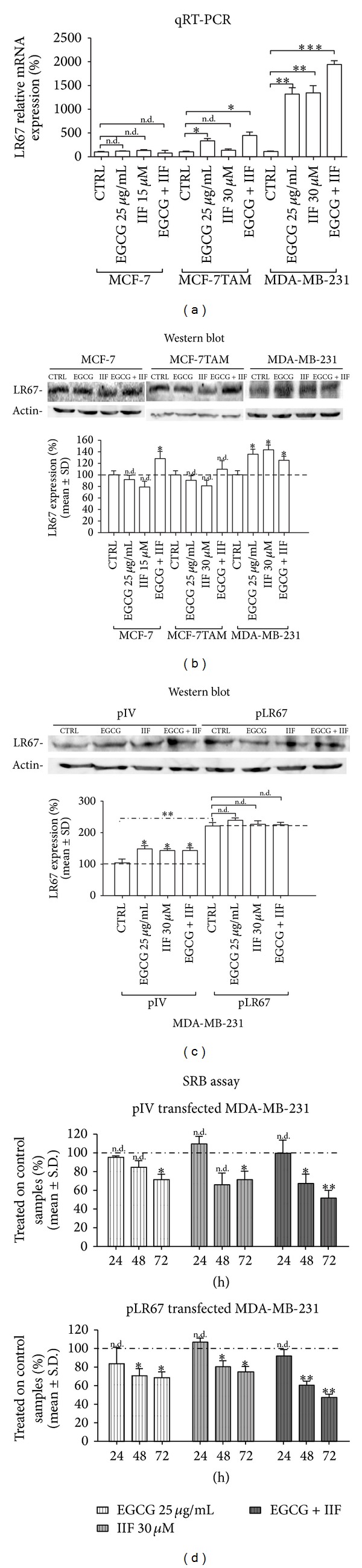
LR67 qRT-PCR and LR67 protein expression. (a) LR67 qRT-PCR: a highly significant increase in LR67 mRNA expression was detected in MDA-MB-231 and MCF-7TAM cells, but not in MCF-7 cells. CTRL: untreated cells. (b) Western blot analysis of LR67: EGCG + IIF treatment was effective in increasing LR67 expression significantly in MCF-7 and MDA-MB-231 cells, whereas a trend was only detected in MCF-7TAM cells. (c) Western blot analysis of LR67 in MDA-MB-231 cells after transfection with vehicle (plV) and pLR67: the significant increase in LR67 protein expression found in MDA-MB-231 cells transfected with pLR67 did not parallel a comparable cell viability decrease (d) in plV and pLR67 transfected MDA-MB-231 cells (SRB assay). *P < 0.05; **P < 0.01; ***P < 0.005. n.d.: not detected as significant.

As shown in Figure 2(a), LR67 mRNA was expressed in MCF-7, MCF-7TAM, and MDA-MB-231 cells. The most significant rise in LR67 expression occurred in MDA-MB-231 cells after all treatments. A modest increase was also detected in MCF-7TAM cells after EGCG and EGCG + IIF treatments. LR67 protein expression increased significantly in MDA-MB-231 cells after all treatments (Figure 2(b)) and in MCF-7 cells treated with EGCG + IIF. LR67 protein was also detected by immunofluorescence in both cytoplasm and plasma membrane in all the cell lines (Supplementary Figures 3A–F).

To define whether LR67 protein increase was determinant in increasing cell cytotoxicity after treatments, we transfected MDA-MB-231 cells with a pCDNA3.1 carrying the LR67 cDNA. LR67 protein expression doubled in positively transfected pLR67 MDA-MB-231 cells (Figure 2(c) and Supplementary Figure 3G-H), but SRB assay did not reveal any significant difference in cell viability between pLR67 and plV MDA-MB-231 cell lines (Figure 2(d)). Then, cytotoxicity was not strictly dependent on LR67 quantity.

### 3.4. Effects of EGCG and IIF Treatments on RXR Expression and Signalling Pathway

**Figure 3:**
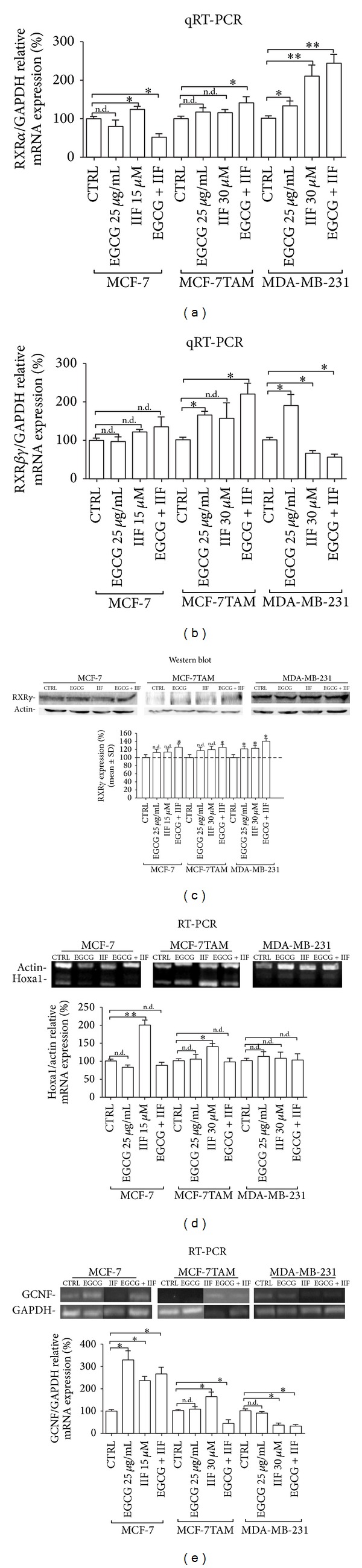
RXR, Hoxa1, and GCNF expression. RXR*α* and RXR*βγ* qRT-PCR ((a) and (b)): MCF-7, MCF-7TAM, and MDA-MB-231 cells responded to treatments increasing RXR*α* and/or *βγ* with the exception of MDA-MB-231 cells, which downregulated RXR*βγ* expression after IIF and EGCG + IIF treatments. (c) RXR*γ* protein Western blot analysis: RXR*γ* protein detection by Western blot. ((d) and (e)) RXR responder gene expression. *P < 0.05; **P < 0.01. n.d.: not detected as significant.

Secondly, we directed our investigation to RXRs, with the aim of evaluating any change in their synthesis and expression. RXRs have three different subtypes (*α*, *β*,*γ*) and loss of RXR*β* and *γ* can be compensated by RXR*α* increase. As we found that RXR*γ* mRNA was hardly detectable (data not shown), we used a primer couple able to cover a homology region in RXR*β* and *γ* genes and we performed qRT-PCR. Remarkably various RXR mRNA expression changes were found in all three cell lines (Figures 3(a)-3(b)), mainly in MCF-7TAM cells after EGCG + IIF treatment. MDA-MB-231 cells responded to treatments by expressing RXR*α* genes. The EGCG + IIF combination was the most effective of all treatments in increasing RXR*γ* protein expression in all the cell lines (Figure 3(c)). EGCG could also upregulate RXR*βγ* expression in MCF-7TAM and MDA-MB-231 cells.

We tried to have a better view of RXR response to treatments in MCF-7, MCF-7TAM, and MDA-MB-231 cells by analysing two RXR responder genes: Homeobox A1 (Hoxa1) and Germ Cell Nuclear Factor (GCNF). Hoxa1 is a “primary response” gene target of retinoic acid, as it possesses enhancers containing a retinoic acid response element (RARE) to which the RXR/RAR heterodimer can bind. At a later time, many genes are transcriptionally regulated and they activate “secondary” target genes such as GCNF, an orphan nuclear receptor. GCNF regulates early embryonic development and it represses octamer-binding transcription factor 4 (Oct-4), a gatekeeper gene able to prevent embryo stem cell differentiation. IIF upregulated Hoxa1 expression in MCF-7 and MCF-7TAM cells. A consistent upregulation of Hoxa1 transcription was induced by IIF treatment in both MCF-7 and MCF-7TAM cells (Figure 3(d)).

We also analysed GCNF expression after 24 h treatments (Figure 3(e)). GCFN expression was present in all the investigated cell lines and it was upregulated in MCF-7 cells, mainly after EGCG + IIF administration. IIF treatment increased GCNF expression in MCF-7TAM cells, whereas EGCG + IIF downregulated GCNF transcription. In contrast, MDA-MB-231 cells showed GCNF downregulation after IIF + EGCG and IIF treatments.

### 3.5. AKT and p473AKT Expression and Foxo3a Immunolocalization

The increased cytotoxicity triggered by the combination of EGCG and IIF could not only be explained by the increase of LR67 and/or RXRs: we considered that EGCG and IIF could have a final common signalling pathway leading to apoptosis that might be strengthened by the individual contributions. We considered AKT signalling pathway and Foxo3a transcription factor good candidates for this explanation. AKT has a strong and well-demonstrated antiapoptotic activity in a wide range of human cancers and it is a potential pharmacological target of therapy in numerous human carcinomas including breast carcinoma. Foxo3a can be inactivated and sequestered in the cytoplasm by AKT activation. When AKT is inactive, Foxo3a is released from the cytoplasm and it goes back to the nucleus, where it promotes apoptosis.

**Figure 4:**
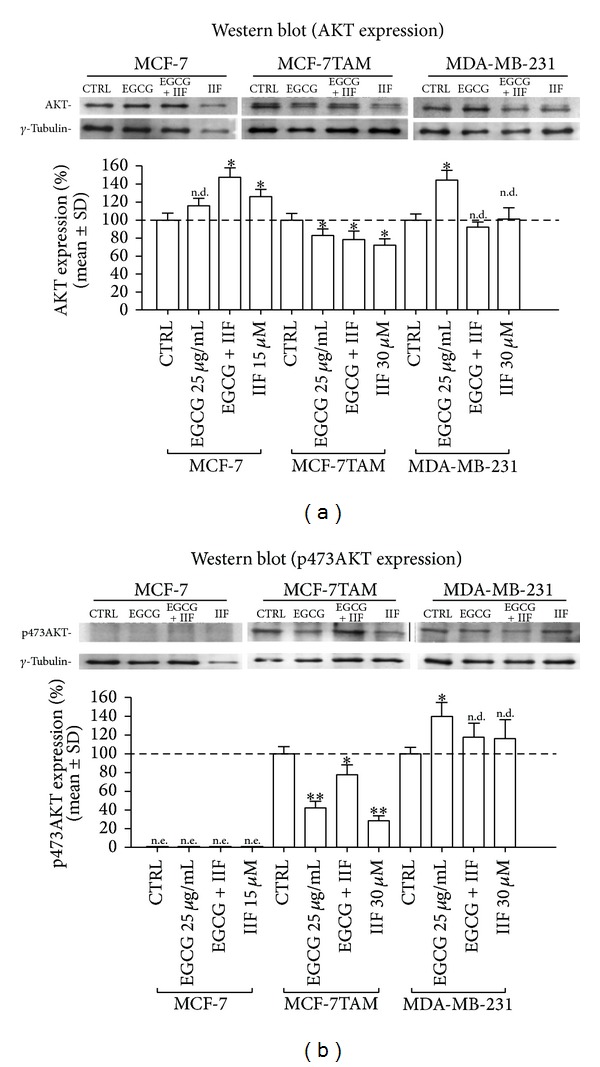
AKT and p473AKT protein expression in MCF-7, MCF-7TAM, and MDA-MB-231 cells. MCF-7TAM cells only showed total AKT (a) and p473AKT protein expression decrease (b) after treatments. MCF-7 cells could not be evaluated as the band corresponding to p473AKT was not revealed. CTRL: untreated cells. *P < 0.05; **P < 0.01. n.d.: not detected as significant.

AKT protein was expressed in all the investigated cell lines: AKT expression increased in MCF-7 cells after EGCG + IIF treatment and in MDA-MB-231 cells after EGCG treatment (Figure 4). In contrast, all the treatments downregulated AKT in MCF-7TAM cells. p473AKT was dramatically reduced in MCF-7TAM cells (Figure 4(a)), whereas we did not detect any band corresponding to p473AKT neither in control nor in treated MCF-7 cells. A significant increase in p473AKT was instead detected in MDA-MB-231 cells after EGCG treatment (Figure 4(b)).

**Figure 5:**
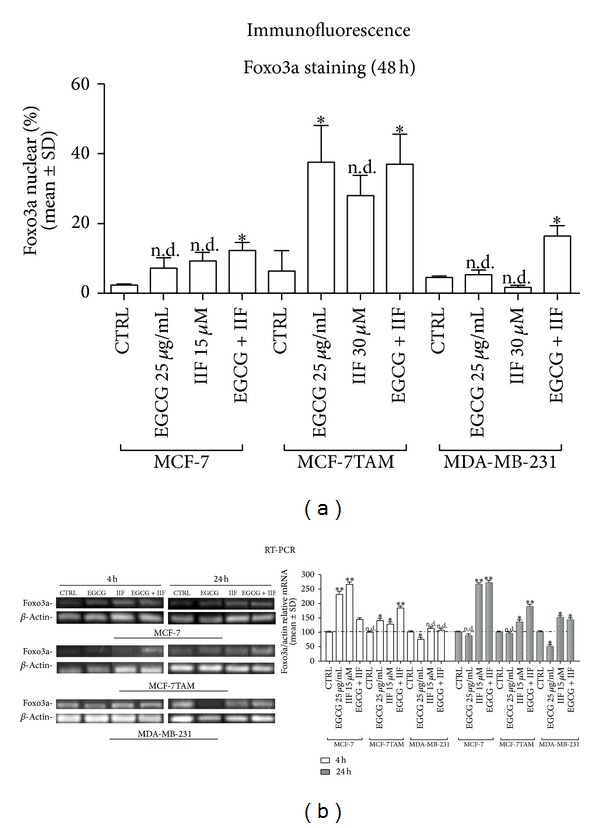
Foxo3a immunolocalization and expression in MCF-7, MCF-7TAM, and MDA-MB-231 cells. (a) Immunofluorescence localization of Foxo3a: Foxo3a was found in the nucleus after EGCG+IIF treatments in all the cell lines, especially in MCF-7TAM cells. *P < 0.05; **P < 0.01. (b) Foxo3a RT-PCR: Foxo3a expression increased after short time treatments (4 h) in MCF-7 and MCF-7TAM cells and at a later time (24 h) in MDA-MB-231 cells. *P < 0.05; **P < 0.01. n.d.: not detected as significant.

Foxo3a was detected by immunofluorescence in the cytoplasm of control cells. Immunostaining was hardly detectable in MDA-MB-231 untreated cells (Supplementary Figure 4). In treated samples, the number of positively stained nuclei increased (Supplementary Figure 4), as well as the fluorescence intensity. MCF-7TAM cells showed a threefold increase in positively stained nuclei after EGCG and EGCG+IIF treatments (Figure 5(a)).

We therefore found that all the three cell lines showed a significant Foxo3a shift, only related to AKT inactivation in MCF-7TAM cells. We considered that other mechanisms might intervene in Foxo3a nuclear positioning and we investigated Foxo3a mRNA expression by RT-PCR after short (4 h) and long (24 h) time treatment (Figure 5(b)). Sustained Foxo3a expression was found in MCF-7 and MCF-7TAM cells after 4 and 24 h treatment, especially after EGCG + IIF administration. In contrast, a significant increase in Foxo3a expression was found in MDA-MB-231 cells only after 24 h treatment.

## 4. Discussion

The rationale of combining agents with different mechanisms of action to increase efficacy on complementary molecular targets and to limit side effects is a current strategy in breast cancer chemoprevention and therapy. In the present study, we explored the cytotoxicity of EGCG, the most important catechin of green tea, and IIF, a RXR ligand under investigation in cancer therapy, given individually and in combination with three breast carcinoma cell lines: MCF-7, MCF-7TAM, and MDA-MB-231 cells.

So far, various chemotherapeutics have been successfully administrated together with EGCG to increase cytotoxicity toward cancer cells: tamoxifen [32], Iressa [33], gemcitabine and CP690550 [34], COX-2 inhibitors, and retinoids including ATRA [35] and cisplatin [36]. Most drugs used in cancer therapy show strong side effects and/or damage to normal cells. We used a combination of molecules demonstrated to be safe for normal cells, either when administered individually or together. In the present study, we showed that EGCG and IIF given at concentrations able to trigger cell death in breast cancer cells did not have significant cytotoxic effects on PHA stimulated human normal lymphocytes.

Cytotoxicity was not only dependent on the increased expression of LR67 or RXR signalling pathway activation. Moreover, Foxo3a, a molecule able to trigger apoptosis, was also activated.

The rationale of the present study was to combine a RXR agonist to EGCG in order to verify whether it could increase LR67 expression and EGCG efficacy as previously demonstrated using EGCG and ATRA [9, 12–14]. Actually, we found that LR67 upregulation was not correlated with increase in cytotoxic effect. The most consistent increase in LR67 expression was detected in MDA-MB-231 cells after all treatments and it was not paralleled by a corresponding cell viability decrease, even after transfection of LR67 cDNA, which doubled LR67 protein amount in MDA-MB-231 cells. On the contrary, MCF-7 cells, which were sensitive to EGCG + IIF treatment, did not significantly show changes in LR67 RNA and protein expression. So we concluded that the increase in LR67 expression was not the main mechanism underlying cytotoxicity when EGCG and IIF were given together. Tachibana and coworkers found an increased expression of LR67 after ATRA treatment in MCF-7 cells, but they did not investigate the effects on MCF-7 or other breast carcinoma cell growth [12]. As we used the RXR agonist IIF together with EGCG, the resulting cytotoxicity ought to be explained by a different mechanism of action. It is also well known that EGCG does not have a single molecular target, but it can trigger apoptosis in cancer cells by modulating a great number of molecules [7]. The final effect was also related to the biomolecular context of the cell line under investigation. We and others have previously demonstrated that EGCG can downregulate ER*α* expression in MCF-7 cells [26] and EGFR in MCF-7TAM cells [37], events that concur to elicit final apoptosis.

Cytotoxicity could not also be completely attributed to RXR signalling activation. IIF is considered RXR*γ* specific [34] but when RXR*γ* is scarcely expressed in a tissue, its function can be replaced by RXR*α* and *β* [38]. RXR isotypes are overexpressed in 66% of breast ductal carcinoma in situ lesions [39] and RXR*α* upregulation was found to be associated with malignant transformation [40]. We found that RXR*βγ* gene expression was balanced by RXR*α* decrease. We also found that EGCG could elicit RXR*βγ* expression after individual treatment in MCF-7TAM and MDA-MB-231 cells. Indirect action of EGCG on the RAR/RXR pathway was demonstrated by Fang and coworkers [41] who found that EGCG could reactivate RAR inhibiting 5-cytosine DNA methyltransferase in human colon, esophageal, and prostate cancer cells, and it could restore responsiveness to RA. Furthermore, along with RAR*β*_2_ activation, ERK1/2/AP1/COX2 were downregulated, resulting in esophageal cancer cell growth arrest and loss of invasivity [42]. In the present study MCF-7TAM and MDA-MB-231 cells had detectable RXR*βγ* expression that was upregulated by EGCG treatment. This finding contributes to pinpoint EGCG as a good molecular partner in course of retinoid/rexinoid treatments. Altogether these data indicated that the RA signalling was triggered variously in all the three cell lines by different mechanisms, although it was not the unique responsibility of the increased cytotoxicity after EGCG+IIF treatment.

The analysis of the retinoid early responder gene Hoxa1 [43] and secondary responder gene GCNF confirmed this hypothesis. We found that IIF only elicited a remarkable Hoxa1 upregulation and GCNF expression in MCF-7 and MCF-7TAM cells, whereas MDA-MB-231 cells did not respond. The interpretation of data concerning GCNF is limited by the scarce information available about the role of GCNF in human cancer cells. GCNF was expressed in 40% of human breast carcinomas [44], but the impact of this finding is far to be explained. We cannot exclude that GCNF is implicated in loss of differentiation in cancer cell and may be subordinated to cancer signalling pathways other than retinoic acid (RA). MDA-MB-231 cells were less responsive to IIF than MCF-7 and MCF-7TAM in terms of increased expression of RXR*βγ* and they showed a prevalent RXR*α* response. MDA-MB-231 cell line is considered representative of the TNBCs, which do not express neither ER*α* nor HER2 [45]. Patients with TNBCs are currently the subgroup of breast carcinoma patients with the worst outcome. TNBCs, however, comprise a wide spectrum of lesions, far to be completely characterized. In the present study, final cytotoxicity was achieved by EGCG and IIF treatment, but the molecular pathway able to activate Foxo3a needs to be better investigated.

We detected Foxo3a in the cytoplasm of control samples and an increasing amount of nuclei showing Foxo3a in treated samples. Foxo3a is a transcription factor directly inactivated by AKT, which is critical for the regulation of cell cycle arrest, cell death, and DNA damage repair [46, 47]. Inactivation of Foxo proteins may be associated with tumorigenesis in breast and prostate carcinoma, glioblastoma, rhabdomyosarcoma, and leukaemia [46]. The present data support a role of Foxo3a as a final molecule downstream EGCG and IIF activity in all the three investigated cell lines, although with different mechanism of activation. In MCF-7TAM cells, Foxo3a change of localisation and activation might be related to AKT inactivation. In tamoxifen resistant cells, AKT activation and EGFR high expression are considered important signalling pathways that sustain proliferation and survival [48]. We have previously demonstrated that EGFR signalling pathway in MCF-7TAM resistant cells was significantly impaired by EGCG, in a dose-dependent manner, by reduced EGFR phosphorylation at tyrosine 1068 [37]. Nuclear Foxo3a shift after treatments occurred in MCF-7 and MDA-MB-231 cells independent of AKT inactivation, but the molecular mechanisms underlying this event need to be further investigated. Both EGCG and retinoids have been demonstrated to activate Foxo3a: in Trastuzumab-resistant HER2-driven breast cancer cells [49] and in ER*α*-positive breast cancer cells [50] the activation of Foxo3a by EGCG played an important role in the resulting cytotoxicity: all samples treated with EGCG + IIF and IIF alone for 24 h showed a significant increase in Foxo3a RNA expression. Retinoids could switch Foxo3a to the nucleus and trigger apoptosis: Foxo3a is a key molecule in ATRA action on acute promyelocytic leukaemia cells [51] as well as RA and IFN-*α* in mantle cell lymphoma [52]. The different mechanisms underlying Foxo3a activation might be related to the different biomolecular characteristics of MCF-7, MCF-7TAM, and MDA-MB-231 cells.

## 5. Conclusions

We found that EGCG and IIF treatments were cytotoxic to breast carcinoma cell lines differing in biomolecular features, including two cell lines representative of unresponsive breast carcinomas. These findings are greatly encouraging to experiment the combination of EGCG and IIF, two safe molecules devoid of toxic effects, in animals and humans, in view of a therapeutic use in human breast cancer.

## Supplementary Material

Supplementary Figure 1: Cytotoxicity assay of EGCG and IIF individual treatments on MCF-7, MCF-7TAM and MDA-MB-231 cells.
Supplementary Figure 2: Apoptosis in PHA stimulated Peripheral Blood Lymphocytes treated with EGCG and IIF.
Supplementary Figure 3: LR67 Immunostaining. LR67 expression was clearly visible in the cytoplasm and plasma membrane of CTRL and treated cells (a-f).
Supplementary Figure 4: Foxo3a immunostaining.
Supplementary Table 1: EGCG and IIF interactions.

## Abbreviations

EGCG: (−)-Epigallocatechin-3-gallate
IIF: 6-OH-11-O-Hydroxyphenanthrene
LR67: 67-kDa laminin receptor
RXRs: Retinoic X receptors
Foxo3a: Forkhead box O3
SERMs: Selective oestrogen receptor modulators
ATRA: All-trans-retinoic acid
AL: Acute leukaemia
RARs: Retinoic acid receptors
ER: Oestrogen receptor
SRB: Sulforhodamine B
pLR67: pCDNA3.1 expressing LR67cDNA
pLV: pCDNA3.1 plasmid vehicle
Hoxa1: Homeobox protein Hox-A1
GCNF: Germ cell nuclear factor.

## References

[1] Wolfram S (2007). Effects of green tea and EGCG on cardiovascular and metabolic health. *Journal of the American College of Nutrition*.

[2] Clement Y (2009). Can green tea do that? A literature review of the clinical evidence. *Preventive Medicine*.

[3] Yuan JM, Sun C, Butler LM (2011). Tea and cancer prevention: epidemiological studies. *Pharmacological Research*.

[4] Kanwar J, Taskeen M, Mohammad I, Huo C, Chan TH, Dou QP (2012). Recent advances on tea polyphenols. *Frontiers in Bioscience*.

[5] Singh BN, Shankar S, Srivastava RK (2011). Green tea catechin, epigallocatechin-3-gallate (EGCG): mechanisms, perspectives and clinical applications. *Biochemical Pharmacology*.

[6] Yang CS, Wang H, Li GX, Yang Z, Guan F, Jin H (2011). Cancer prevention by tea: evidence from laboratory studies. *Pharmacological Research*.

[7] Yang CS, Wang H (2011). Mechanistic issues concerning cancer prevention by tea catechins. *Molecular Nutrition and Food Research*.

[8] Henning SM, Niu Y, Liu Y (2005). Bioavailability and antioxidant effect of epigallocatechin gallate administered in purified form versus as green tea extract in healthy individuals. *Journal of Nutritional Biochemistry*.

[9] Lee JH, Kishikawa M, Kumazoe M, Yamada K, Tachibana H (2010). Vitamin A enhances antitumor effect of a green tea polyphenol on melanoma by upregulating the polyphenol sensing molecule 67-kDa laminin receptor. *PLoS ONE*.

[10] Mi J (2011). Current treatment strategy of acute promyelocytic leukemia. *Frontiers of Medicine in China*.

[11] Nelson J, Mcferran NV, Pivato G (2008). The 67 kDa laminin receptor: structure, function and role in disease. *Bioscience Reports*.

[12] Tachibana H, Koga K, Fujimura Y, Yamada K (2004). A receptor for green tea polyphenol EGCG. *Nature Structural and Molecular Biology*.

[13] Umeda D, Yano S, Yamada K, Tachibana H (2008). Green tea polyphenol epigallocatechin-3-gallate signaling pathway through 67-kDa laminin receptor. *Journal of Biological Chemistry*.

[14] Umeda D, Yano S, Yamada K, Tachibana H (2008). Involvement of 67-kDa laminin receptor-mediated myosin phosphatase activation in antiproliferative effect of epigallocatechin-3-O-gallate at a physiological concentration on Caco-2 colon cancer cells. *Biochemical and Biophysical Research Communications*.

[15] Anzano MA, Byers SW, Smith JM (1994). Prevention of breast cancer in the rat with 9-cis-retinoic acid as a single agent and in combination with tamoxifen. *Cancer Research*.

[16] Anzano MA, Peer CW, Smith JM (1996). Chemoprevention of mammary carcinogenesis in the rat: combined use of raloxifene and 9-cis-retinoic acid. *Journal of the National Cancer Institute*.

[17] Bryan M, Pulte ED, Toomey KC (2011). A pilot phase II trial of all-trans retinoic acid (Vesanoid) and paclitaxel (Taxol) in patients with recurrent or metastatic breast cancer. *Investigational New Drugs*.

[18] Budd GT, Adamson PC, Gupta M (1998). Phase I/II trial of all-trans retinoic acid and tamoxifen in patients with advanced breast cancer. *Clinical Cancer Research*.

[19] Cazzaniga M, Varricchio C, Montefrancesco C, Feroce I, Guerrieri-Gonzaga A (2012). Fenretinide (4-HPR): a preventive chance for women at genetic and familial risk?. *Journal of Biomedicine and Biotechnology*.

[20] Wu K, Zhang Y, Xu X-C (2002). The retinoid X receptor-selective retinoid, LGD1069, prevents the development of estrogen receptor-negative mammary tumors in transgenic mice. *Cancer Research*.

[21] Uray IP, Brown PH (2011). Chemoprevention of hormone receptor-negative breast cancer: new approaches needed. *Recent Results in Cancer Research*.

[22] Bartolini G, Orlandi M, Papi A (2006). A search for multidrug resistance modulators: the effects of retinoids in human colon carcinoma cells. *In Vivo A*.

[23] Papi A, Rocchi P, Ferreri AM, Orlandi M (2010). RXR*γ* and PPAR*γ* ligands in combination to inhibit proliferation and invasiveness in colon cancer cells. *Cancer Letters*.

[24] Papi A, Guarnieri T, Storci G (2012). Nuclear receptors agonists exert opposing effects on the inflammation dependent survival of breast cancer stem cells. *Cell Death and Differentiation*.

[25] Montesinos P, Sanz MA (2011). The differentiation syndrome in patients with acute promyelocytic leukemia: experience of the pethema group and review of the literature. *Mediterranean Journal of Hematology and Infectious Diseases*.

[26] Farabegoli F, Barbi C, Lambertini E, Piva R (2007). (−)-Epigallocatechin-3-gallate downregulates estrogen receptor alpha function in MCF-7 breast carcinoma cells. *Cancer Detection and Prevention*.

[27] Romanelli S, Perego P, Pratesi G, Carenini N, Tortoreto M, Zunino F (1998). *In vitro* and *in vivo* interaction between cisplatin and topotecan in ovarian carcinoma systems. *Cancer Chemotherapy and Pharmacology*.

[28] Chomczynski P, Sacchi N (1987). Single-step method of RNA isolation by acid guanidinium thiocyanate-phenol-chloroform extraction. *Analytical Biochemistry*.

[29] Kuo CC, Lin SC (2007). Altered FOXO1 transcript levels in peripheral blood mononuclear cells of systemic lupus erythematosus and rheumatoid arthritis patients. *Molecular Medicine*.

[30] Liu L, Sun L, Zhao P (2010). Hypoxia promotes metastasis in human gastric cancer by up-regulating the 67-kDa laminin receptor. *Cancer Science*.

[31] Livak KJ, Schmittgen TD (2001). Analysis of relative gene expression data using real-time quantitative PCR and the 2-ΔΔCT method. *Methods*.

[32] Scandlyn MJ, Stuart EC, Somers-Edgar TJ, Menzies AR, Rosengren RJ (2008). A new role for tamoxifen in oestrogen receptor-negative breast cancer when it is combined with epigallocatechin gallate. *British Journal of Cancer*.

[33] Chang CM, Chang PY, Tu MG (2012). Epigallocatechin gallate sensitizes CAL-27 human oral squamous cell carcinoma cells to the anti-metastatic effects of gefitinib (Iressa) via synergistic suppression of epidermal growth factor receptor and matrix metalloproteinase-2. *Oncology Reports*.

[34] Tang SN, Fu J, Shankar S, Srivastava RK (2012). EGCG enhances the therapeutic potential of gemcitabine and CP690550 by inhibiting STAT3 signaling pathway in human pancreatic cancer. *PLoS ONE*.

[35] Suganuma M, Saha A, Fujiki H (2011). New cancer treatment strategy using combination of green tea catechins and anticancer drugs. *Cancer Science*.

[36] Singh M, Bhui K, Singh R, Shukla Y (2013). Tea polyphenols enhance cisplatin chemosensitivity in cervical cancer cells via induction of apoptosis. *Life Sciences*.

[37] Farabegoli F, Papi A, Orlandi M (2011). (−)-Epigallocatechin-3-gallate down-regulates EGFR, MMP-2, MMP-9 and EMMPRIN and inhibits the invasion of MCF-7 tamoxifen-resistant cells. *Bioscience Reports*.

[38] Lefebvre P, Benomar Y, Staels B (2010). Retinoid X receptors: common heterodimerization partners with distinct functions. *Trends in Endocrinology and Metabolism*.

[39] Lawrence JA, Merino MJ, Simpson JF, Manrow RE, Page DL, Steeg PS (1998). A high-risk lesion for invasive breast cancer, ductal carcinoma in situ, exhibits frequent overexpression of retinoid X receptor. *Cancer Epidemiology Biomarkers and Prevention*.

[40] Friedrich M, Axt-Fliedner R, Villena-Heinsen C, Tilgen W, Schmidt W, Reichrath J (2002). Analysis of vitamin D-receptor (VDR) and retinoid X-receptor *α* in breast cancer. *Histochemical Journal*.

[41] Fang MZ, Wang Y, Ai N (2003). Tea polyphenol (−)-epigallocatechin-3-gallate inhibits DNA methyltransferase and reactivates methylation-silenced genes in cancer cell lines. *Cancer Research*.

[42] Ye F, Zhang G-H, Guan B-X, Xu X-C (2012). Suppression of esophageal cancer cell growth using curcumin, (−)-epigallocatechin-3-gallate and lovastatin. *World Journal of Gastroenterology*.

[43] Gudas LJ, Wagner JA (2011). Retinoids regulate stem cell differentiation. *Journal of Cellular Physiology*.

[44] Bianco C, Castro NP, Baraty C (2013). Regulation of human Cripto-1 expression by nuclear receptors and DNA promoter methylation in human embryonal and breast cancer cells. *Journal of Cellular Physiology*.

[45] Turner NC, Reis-Filho JS (2013). Tackling the diversity of triple-negative breast cancer. *Clinical Cancer Research*.

[46] Yang JY, Hung MC (2009). A new fork for clinical application: targeting forkhead transcription factors in cancer. *Clinical Cancer Research*.

[47] Huang H, Tindall DJ (2006). FOXO transcription factors in cell-cycle regulation and the response to oxidative stress. *Future Oncology*.

[48] Riggins RB, Schrecengost RS, Guerrero MS, Bouton AH (2007). Pathways to tamoxifen resistance. *Cancer Letters*.

[49] Eddy SF, Kane SE, Sonenshein GE (2007). Trastuzumab-resistant HER2-driven breast cancer cells are sensitive to epigallocatechin-3 gallate. *Cancer Research*.

[50] Belguise K, Guo S, Sonenshein GE (2007). Activation of FOXO3a by the green tea polyphenol epigallocatechin-3-gallate induces estrogen receptor *α* expression reversing invasive phenotype of breast cancer cells. *Cancer Research*.

[51] Sakoe Y, Sakoe K, Kirito K, Ozawa K, Komatsu N (2010). FOXO3A as a key molecule for all-trans retinoic acid-induced granulocytic differentiation and apoptosis in acute promyelocytic leukemia. *Blood*.

[52] Col JD, Mastorci K, Fae DA (2012). Retinoic acid/alpha-interferon combination inhibits growth and promotes apoptosis in mantle cell lymphoma through Akt-dependent modulation of critical targets. *Cancer Research*.

