# Supplementary material for: A RXR Ligand 6-OH-11-O-Hydroxyphenanthrene with Antitumour Properties Enhances (−)-Epigallocatechin-3-gallate Activity in Three Human Breast Carcinoma Cell Lines
Source: Biomed Res Int. 2014 Jun 11;2014:853086. doi: 10.1155/2014/853086 (PMC4072039; doi:10.1155/2014/853086)
Supplement: Supplementary file 1 — Supplementary Figure 1: Cytotoxicity assay of EGCG and IIF individual treatments on MCF-7, MCF-7TAM and MDA-MB-231 cells. Supplementary Figure 2: Apoptosis in PHA stimulated Peripheral Blood Lymphocytes treated with EGCG and IIF. Supplementary Figure 3: LR67 Immunostaining. LR67 expression was clearly visible in the cytoplasm and plasma membrane of CTRL and treated cells (a-f). Supplementary Figure 4: Foxo3a immunostaining. Supplementary Table 1: EGCG and IIF interactions. [file 853086.f1.zip › v2/description.docx]

*Supplementary Figure 1:* ***Cytotoxicity assay of EGCG and IIF individual treatments on MCF-7,***

***MCF-7TAM and MDA-MB-231 cells.*** *EGCG cytotoxicity was greater in MCF-7 (A) and MCF-*

*7TAM (B) than in MDA-MB-231 cells (C). In contrast, IIF cytotoxicity was lower in MCF-7 (D)*

*and MCF-7TAM (E) than in MDA-MB-231 cells (F). Cells were grown in 96-well-plates and*

*incubated with 0, 25, 50, 100 μg/ml EGCG and 0, 10, 20, 30, 40 μM IIF for 24, 48 and 72 h. Cell*

*viability was measured by SRB assay. Data shown represent the % treated on control samples of*

*three replicas confirmed in two independent experiments. * p< 0.05; ** p<0.01. n.d. Not detected*

*as significant.*

*Supplementary Table 1:* ***EGCG and IIF interactions.*** *SRB assay data (Fig.1A) were used to*

*calculate additivity (=1), synergism (>1) or antagonism (<1) after EGCG and/or IIF treatments at*

*the following concentrations: EGCG 25* μ*g/ml, IIF 15* μ*M (MCF-7) and 30* μ*M (MCF-7TAM and*

*MDA-MB-231).*

*Supplementary Figure 2:* ***Apoptosis in PHA stimulated Peripheral Blood Lymphocytes treated***

***with EGCG and IIF.*** *Normal cells did not show a significant increase in apoptotic nuclei after*

*treatments. Methanol fixation and DAPI staining after 72 h incubation with EGCG, IIF and*

*EGCG+IIF at the indicated concentrations. The number of apoptotic nuclei for field was recorded*

*by epifluorescence microscope and the average percentage was calculated. At least 150 cells were*

*scored for sample. *p< 0.05; **p<0.01. n.d. Not detected as significant.*

*Supplementary Figure 3:* ***LR67 Immunostaining.*** *LR67 expression was clearly visible in the*

*cytoplasm and plasma membrane of CTRL and treated cells (a-f). LR67 immunostaining*

*remarkably increased in MDA-MB-231 cells after transfection with pLR67 (h) with respect to cells*

*transfected with vehicle (plV, g). Cells were fixed in 1% Formalin and incubated with anti-LR67*

*primary antibody and anti-mouse-FITC secondary antibody as described in Section 2.8. *p< 0.05;*

***p<0.01. n.d. Not detected as significant.*

*Supplementary Figure 4:* ***Foxo3a immunostaining.*** *Foxo3a was detectable in the cytoplasm of*

*untreated CTRL cells (a) whereas immunostaining was also detected in a various percentage of*

*nuclei in EGCG+IIF treated cells (c) as reported in the diagram. *p< 0.05; **p<0.01. n.d. Not*

*detected as significant.*
