# Supplementary figures and images for: A RXR Ligand 6-OH-11-O-Hydroxyphenanthrene with Antitumour Properties Enhances (−)-Epigallocatechin-3-gallate Activity in Three Human Breast Carcinoma Cell Lines
Source: Biomed Res Int. 2014 Jun 11;2014:853086. doi: 10.1155/2014/853086 (PMC4072039; doi:10.1155/2014/853086)

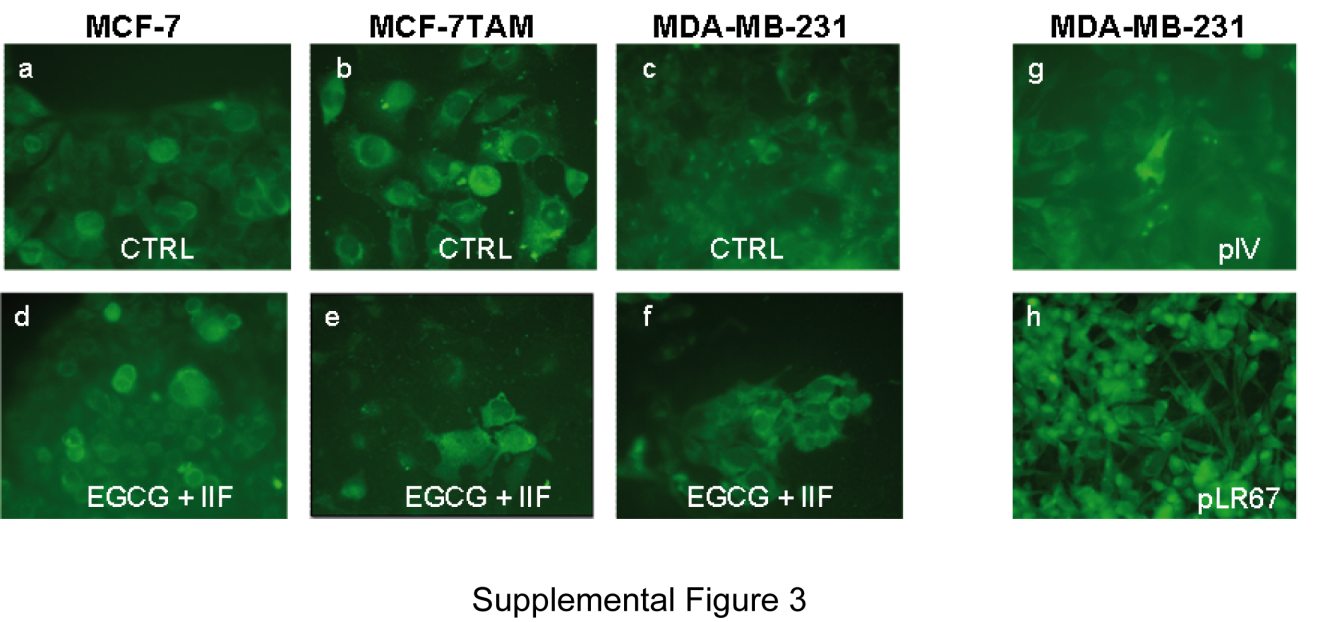

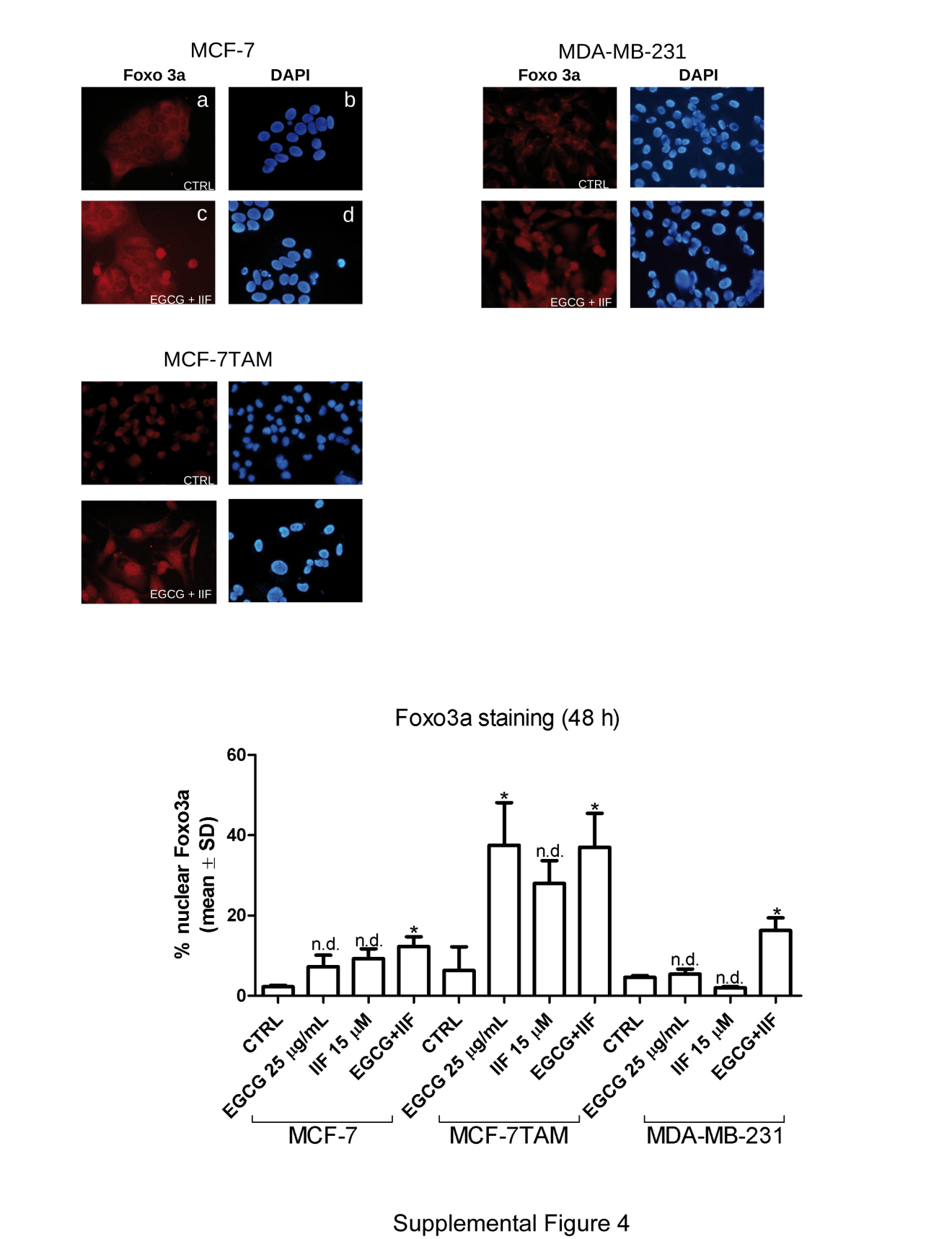

Supplement: Supplementary file 1 — Supplementary Figure 1: Cytotoxicity assay of EGCG and IIF individual treatments on MCF-7, MCF-7TAM and MDA-MB-231 cells. Supplementary Figure 2: Apoptosis in PHA stimulated Peripheral Blood Lymphocytes treated with EGCG and IIF. Supplementary Figure 3: LR67 Immunostaining. LR67 expression was clearly visible in the cytoplasm and plasma membrane of CTRL and treated cells (a-f). Supplementary Figure 4: Foxo3a immunostaining. Supplementary Table 1: EGCG and IIF interactions. [file 853086.f1.zip › v2/Doc1.docx]

A

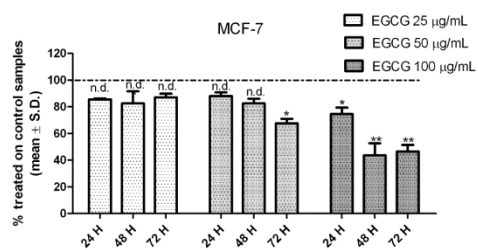

B

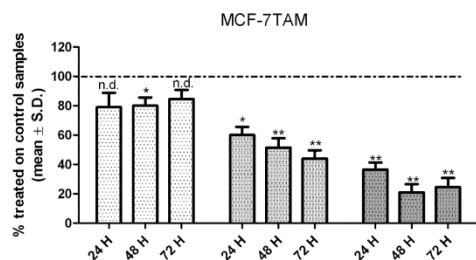

C

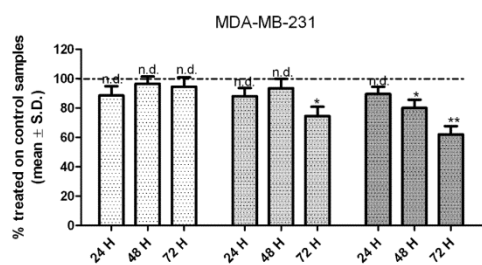

D

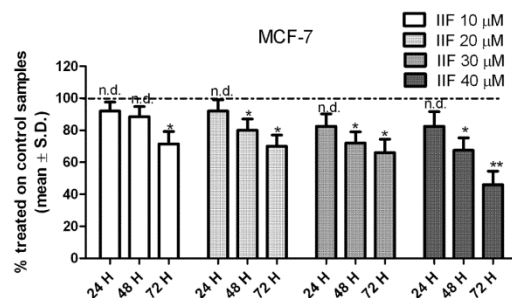

E

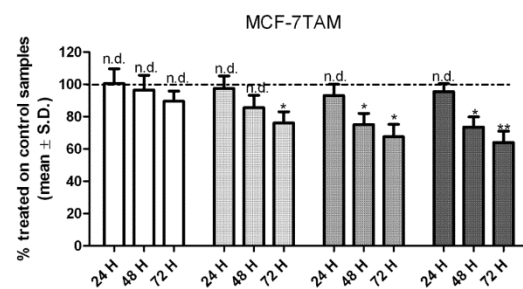

F

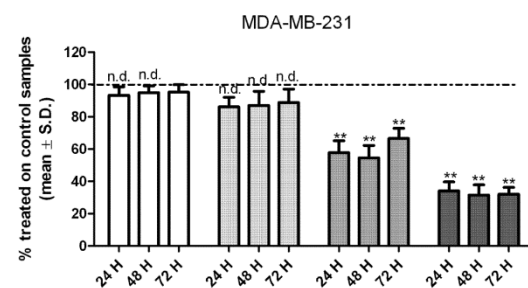

Supplemental Figure 1

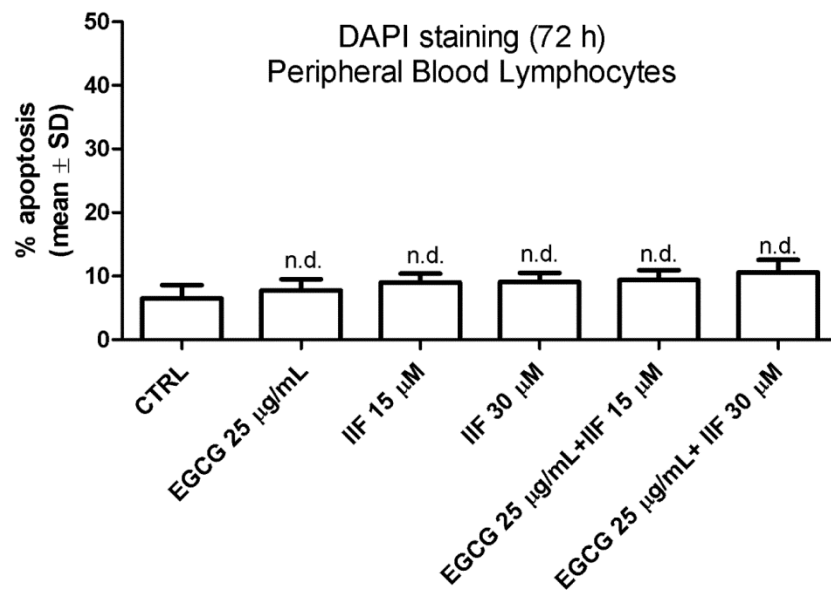

Supplemental Figure 2

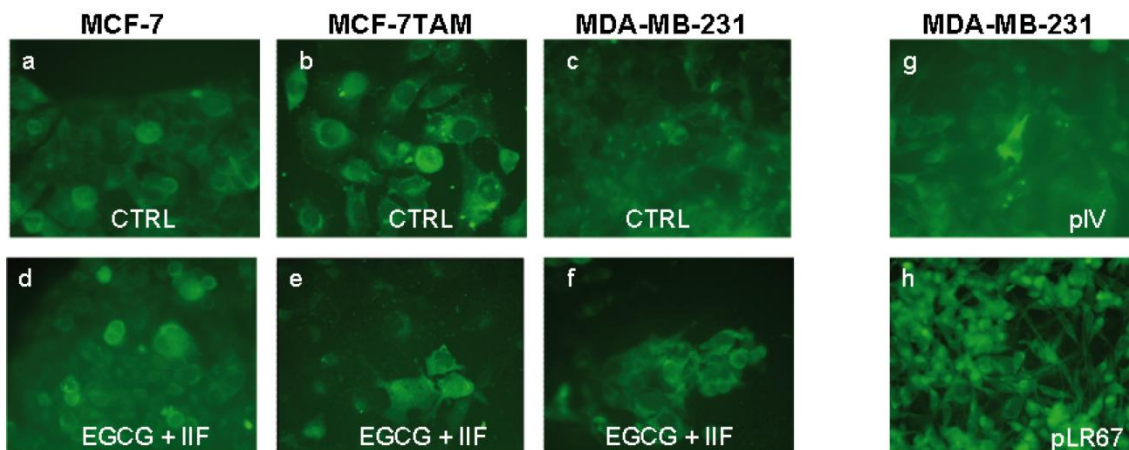

Supplemental Figure 3

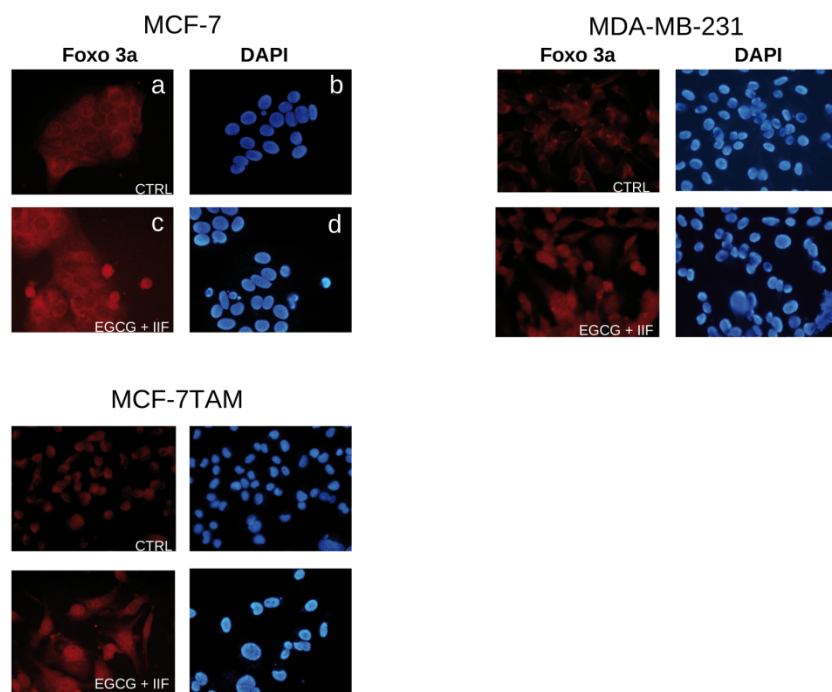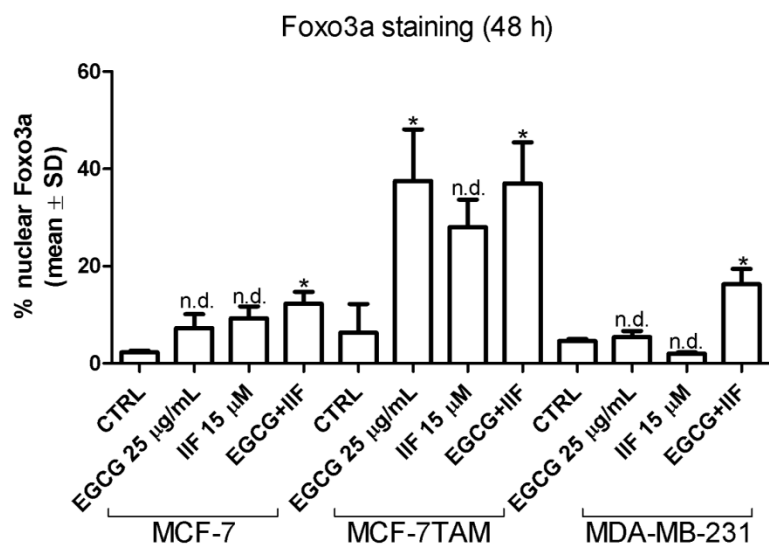

Supplemental Figure 4

Supplement: Supplementary file 1 — Supplementary Figure 1: Cytotoxicity assay of EGCG and IIF individual treatments on MCF-7, MCF-7TAM and MDA-MB-231 cells. Supplementary Figure 2: Apoptosis in PHA stimulated Peripheral Blood Lymphocytes treated with EGCG and IIF. Supplementary Figure 3: LR67 Immunostaining. LR67 expression was clearly visible in the cytoplasm and plasma membrane of CTRL and treated cells (a-f). Supplementary Figure 4: Foxo3a immunostaining. Supplementary Table 1: EGCG and IIF interactions. [file 853086.f1.zip › v2/Doc1.pdf]
