# Supplementary material for: A RXR Ligand 6-OH-11-O-Hydroxyphenanthrene with Antitumour Properties Enhances (−)-Epigallocatechin-3-gallate Activity in Three Human Breast Carcinoma Cell Lines
Source: Biomed Res Int. 2014 Jun 11;2014:853086. doi: 10.1155/2014/853086 (PMC4072039; doi:10.1155/2014/853086)
Supplement: Supplementary file 1 — Supplementary Figure 1: Cytotoxicity assay of EGCG and IIF individual treatments on MCF-7, MCF-7TAM and MDA-MB-231 cells. Supplementary Figure 2: Apoptosis in PHA stimulated Peripheral Blood Lymphocytes treated with EGCG and IIF. Supplementary Figure 3: LR67 Immunostaining. LR67 expression was clearly visible in the cytoplasm and plasma membrane of CTRL and treated cells (a-f). Supplementary Figure 4: Foxo3a immunostaining. Supplementary Table 1: EGCG and IIF interactions. [file 853086.f1.zip › v2/SUPTAB1.doc]

**Table 1. EGCG and IIF interactions**

|  | **24 h** | **48 h** | **72 h** |
| --- | --- | --- | --- |
| **MCF-7** | 1,02 | 1,1 | 1,1 |
| **MCF-7TAM** | 0,99 | 1,01 | 0,68 |
| **MDA-MB-231** | 0,96 | 1,01 | 1,05 |

SRB assay data (Fig.1A) were used to calculate additivity (=1), synergism (>1) or antagonism (<1) after EGCG and/or IIF treatments at the following concentrations: EGCG 25 μg/ml, IIF 15 μM (MCF-7) and 30 μM (MCF-7TAM and MDA-MB-231).
